# Real-time metabolic profiling of oesophageal tumours reveals an altered metabolic phenotype to different oxygen tensions and to treatment with Pyrazinib

**DOI:** 10.1038/s41598-020-68777-7

**Published:** 2020-07-21

**Authors:** Amy M. Buckley, Margaret R. Dunne, Maria E. Morrissey, Susan A. Kennedy, Aoife Nolan, Maria Davern, Emma K. Foley, Niamh Clarke, Joanne Lysaght, Narayanasamy Ravi, Dermot O’Toole, Finbar MacCarthy, John V. Reynolds, Breandán N. Kennedy, Jacintha O’Sullivan

**Affiliations:** 1 Department of Surgery, Trinity Translational Medicine Institute, St. James’s Hospital, Trinity College Dublin, Dublin, Ireland; 2 Department of Clinical Medicine, Trinity Translational Medicine Institute, St. James’s Hospital, Trinity College Dublin, Dublin, Ireland; 3 UCD Conway Institute and UCD School of Biomolecular and Biomedical Science, University College Dublin, Dublin, Ireland

**Keywords:** Phenotypic screening, Oesophageal cancer

## Abstract

Oesophageal cancer is the 6th most common cause of cancer related death worldwide. The current standard of care for oesophageal adenocarcinoma (OAC) focuses on neoadjuvant therapy with chemoradiation or chemotherapy, however the 5-year survival rates remain at < 20%. To improve treatment outcomes it is critical to further investigate OAC tumour biology, metabolic phenotype and their metabolic adaptation to different oxygen tensions. In this study, by using human ex-vivo explants we demonstrated using real-time metabolic profiling that OAC tumour biopsies have a significantly higher oxygen consumption rate (OCR), a measure of oxidative phosphorylation compared to extracellular acidification rate (ECAR), a measure of glycolysis (p = 0.0004). Previously, we identified a small molecule compound, pyrazinib which enhanced radiosensitivity in OAC. Pyrazinib significantly inhibited OCR in OAC treatment-naïve biopsies (p = 0.0139). Furthermore, OAC biopsies can significantly adapt their metabolic rate in real-time to their environment. Under hypoxic conditions pyrazinib produced a significant reduction in both OCR (p = 0.0313) and ECAR in OAC treatment-naïve biopsies. The inflammatory secretome profile from OAC treatment-naïve biopsies is heterogeneous. OCR was positively correlated with three secreted factors in the tumour conditioned media: vascular endothelial factor A (VEGF-A), IL-1RA and thymic stromal lymphopoietin (TSLP). Pyrazinib significantly inhibited IL-1β secretion (p = 0.0377) and increased IL-3 (p = 0.0020) and IL-17B (p = 0.0181). Importantly, pyrazinib did not directly alter the expression of dendritic cell maturation markers or reduce T-cell viability or activation markers. We present a new method for profiling the metabolic rate of tumour biopsies in real-time and demonstrate the novel anti-metabolic and anti-inflammatory action of pyrazinib ex-vivo in OAC tumours, supporting previous findings in-vitro whereby pyrazinib significantly enhanced radiosensitivity in OAC.

## Introduction

Dysregulated cellular energetics is an emerging hallmark of cancer whereby cancer cells adapt to sustain rapid growth and proliferation through altering their metabolism. The first tumour-specific alteration in metabolism was reported by Otto Warburg^1^. Warburg demonstrated that cancer cells relied on an increased glycolytic flux, whereby cancer cells were found to constitutively take up glucose even in the presence of oxygen, an effect which is now commonly referred to as “aerobic glycolysis” or the “Warburg’s effect”^1^. The increase in glycolytic flux was thought to fulfil the metabolic demands of proliferating cells^2^. Whilst the “Warburg effect” has been repeatedly reported in multiple studies, it is well established in recent years that cancer cells also require mitochondrial respiration^3^. There is now accumulating evidence in the literature that suggests certain subpopulations of tumour cells exhibited effects of a high dependence on oxidative phosphorylation and reduced glycolytic dependence^4^. Examples of such populations are stem-like sub-clones and circulating tumour cells (CTCs)^5^. In pancreatic ductal carcinoma xenografts, the cancer stem cells responsible for tumour relapse had increased oxidative phosphorylation and mitochondrial biogenesis, furthermore, relapsing tumours were responsive to the mitochondrial complex V inhibitor, oligomycin^5^. In MCF-7 breast cancer stem cells, the mitochondrial complex III inhibitor, atovaquone, inhibited cancer stem-cell proliferation through the inhibition of oxygen-consumption^6^. OAC is an aggressive disease with a poor 5-year survival rate of < 20%. Both oxidative phosphorylation and inflammation are linked with radiation resistance in OAC^7–9^. We previously demonstrated the important role of oxidative phosphorylation in response to neoadjuvant therapy treatment in OAC^10^. Interestingly, using an in-vitro model of OAC radioresistance, an altered metabolic phenotype exists in which radioresistant cells have a significantly higher oxygen consumption rate (OCR), compared to radiation sensitive cells^10^. Furthermore, in treatment-naïve OAC patient samples ATP5B, a marker of oxidative phosphorylation was present at significantly higher levels in the biopsies of OAC patients who had a subsequent poor response to neoadjuvant chemoradiation therapy (neoCRT), compared to patients who had a good response^10^. Targeting oxidative phosphorylation with a pyrazine compound, pyrazinib (P3) enhanced radiosensitivity in-vitro in an isogenic model of OAC radioresistance^7^. To date however, metabolic rate has not yet been assessed in real-time in OAC biopsies ex vivo. Understanding the real-time metabolic rate of human OAC treatment-naïve biopsies would provide a novel and detailed insight into the metabolic phenotype of OAC tumours to identify potential mechanisms of treatment resistance. Furthermore, by assessing metabolism in real-time ex vivo*,* there exists an added opportunity to evaluate novel anti-metabolic agents in this human ex vivo model system. In this study we sought to investigate for the first time the real-time metabolic rate of treatment-naïve OAC tumour biopsies, under both conditions of normoxia and hypoxia, and to evaluate the potential effect of pyrazinib (P3) on the metabolic rate of treatment-naïve OAC biopsies ex vivo.

Inflammation and metabolism are tightly linked biological processes as inflammatory cells rely on metabolites generated from the metabolic cycle to maintain their function. OAC is an inflammation-driven cancer and inflammation acts as a negative regulator of neoadjuvant treatment response in OAC^11,12^. Circulating levels of the inflammatory mediator leukaemia inhibitory factor in pre-treatment serum of OAC patients is higher in patients with a subsequent poor pathological response to neoadjuvant treatment^8^. Additionally, C3a and C4a, components of the complement system are upregulated in the pre-treatment serum of OAC patients having a subsequent poor pathological response to neoCRT, compared to patients having a favourable response to treatment^12^. In this study we characterised the inflammatory secretion profiles from 22 OAC patient tumours and correlated this with matched real-time metabolic profiles to further understand the tumour biology and role of supporting tumour microenvironment in OAC in these patient tumours.

Furthermore, solid malignancies can trigger an intrinsic inflammatory response that establishes a pro-tumorigenic microenvironment, but this can also lead to the host mounting an anti-tumoural response. Anti-tumoural immunity relies on the function of key immune cells including dendritic cells and effector T cells to eradicate tumour cells, thus it is critical in the development of new therapeutics to ensure that such compounds do not negatively alter the function of anti-tumour immune cells^13^. In this study we also investigated the effect of the anti-metabolic agent pyrazinib (P3) on the expression of dendritic cell maturation markers and T cell viability and activation.

In this study for the first time we demonstrate the real-time metabolic profiles in OAC tumours whereby they display a significantly higher oxygen consumption rate (OCR), a measure of oxidative phosphorylation compared to extracellular acidification rate (ECAR), a measure of glycolysis highlighting the importance of aerobic respiration in OAC. Critically, biopsies from OAC tumours can adapt their metabolic rate to their environment to cope with hypoxia. Under normoxic conditions, pyrazinib (P3), a dual action small molecule radiosensitiser significantly inhibited OCR in OAC biopsies. Under hypoxic conditions, pyrazinib (P3) significantly reduced OCR and ECAR in OAC treatment-naïve biopsies. Furthermore, the inflammatory secretion profile from OAC treatment-naïve biopsies is highly heterogeneous. OCR positively correlated with three secreted factors in OAC tumour conditioned media: VEGF-A, IL-1RA and TSLP. ECAR was positively correlated with VEGF-A, IL-1RA, TSLP, IL-13, MIP-3α and tumour necrosis factor alpha (TNF-α). Pyrazinib (P3) significantly inhibited IL-1β secretion and increased IL-3 and IL-17B secretion from treatment-naïve biopsies from OAC patients. Critically, pyrazinib (P3) does not negatively affect dendritic cell function or T cell viability or activation marker expression. We present a new method for profiling the metabolic rate in OAC tumour biopsies in real-time and demonstrate the significant correlation OCR with the secreted levels of VEGF-A, IL-1RA and TSLP. Furthermore, we demonstrate the novel anti-metabolic and anti-inflammatory action of pyrazinib (P3) ex vivo in OAC treatment-naïve biopsies.

## Results

### Oxidative Phosphorylation was significantly higher than glycolysis in OAC treatment-naïve biopsies

**Figure 1:**
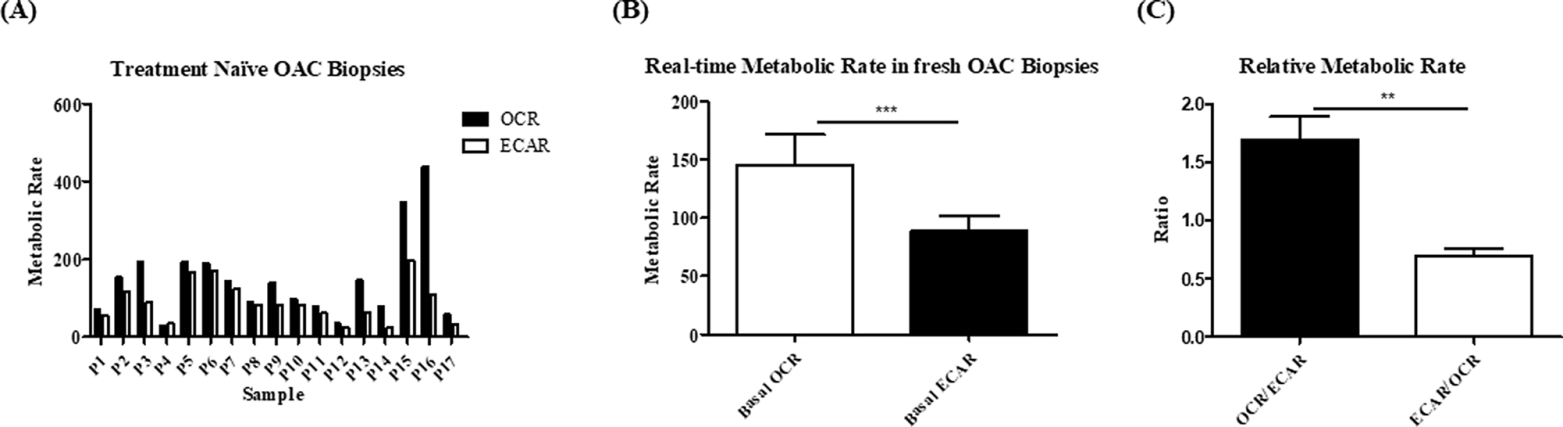
Oxygen consumption rate is significantly higher than extracellular acidification rate in OAC treatment-naïve biopsies. OCR, a measure of oxidative phosphorylation and ECAR, a measure of glycolysis were assessed in real-time in OAC treatment-naïve biopsies using the Seahorse Biosciences XFe24 Analyser. (**A**) Basal OCR and ECAR rates in OAC pre-treatment biopsies, (n = 17). (**B**) OCR is significantly elevated in OAC treatment-naïve biopsies, (n = 17), Wilcoxon signed rank test, ***p < 0.001. (**C**) Relative metabolic ratio OCR:ECAR compared to ECAR:OCR in OAC treatment naïve biopsies (n = 17), paired t-test, **p < 0.01. Data expressed as + SEM.

Clinical patient characteristics are outlined in Supplemental Table 1 and Supplemental Table 2. In this study we investigated the real-time metabolic rate to understand the importance of aerobic respiration in 17 fresh OAC biopsies using the Seahorse Biosciences XFe24 analyser. Real-time metabolic profiling of OAC pre-treatment biopsies from 17 patients demonstrated that biopsies from OAC treatment-naïve tumours have a significantly higher OCR, a measure of oxidative phosphorylation, compared to ECAR, a measure of glycolysis (*p* = 0.0005), highlighting the importance of aerobic respiration in OAC tumours (Fig. 1A,B). The rate of OCR and ECAR was heterogeneous across our patient cohort. In addition, the relative ratio of OCR:ECAR was significantly higher than the relative rate of ECAR:OCR (Fig. 1C), (*p* = 0.0013) further highlighting the importance of oxidative phosphorylation in OAC treatment-naïve biopsies. To investigate the relationship between real-time metabolic rate and clinical patient characteristics, OCR and ECAR were divided according to nodal status, tumour stage, stage of differentiation, body mass index, age at diagnosis and gender of the patients evaluated in this study. OCR and ECAR were shown to be independent of clinical patient characteristics, whereby there was no significant difference in the levels of OCR or ECAR when assessed according to patient characteristics (Supplemental Fig. 1, 2). All metabolic readouts were normalised to biopsy protein content following this assay; thus a limitation of this study was biopsy fragments directly adjacent to the fragment used in study had to be used to confirm the pathology of the tumour.

In summary, OAC treatment-naïve biopsies have a significantly higher rate of OCR than ECAR and real-time metabolic rate is not significantly associated with clinical patient characteristics, which indicates that oxidative phosphorylation is an important metabolic pathway active across all n = 17 OAC tumours evaluated in this study, regardless of clinical patient characteristics.

### Pyrazinib (P3) significantly inhibited oxygen consumption rate in OAC treatment-naïve biopsies

**Figure 2:**
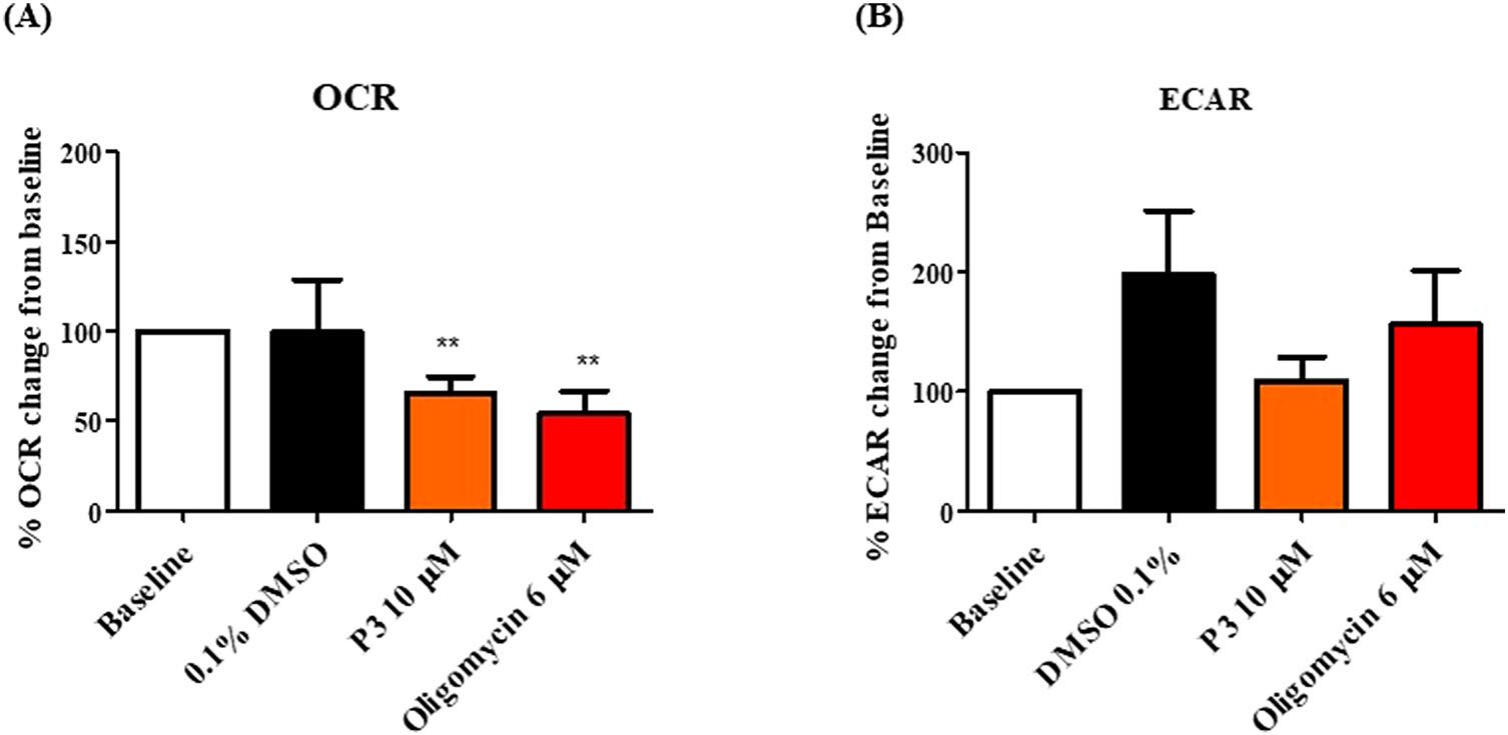
Pyrazinib (P3) significantly reduces OCR in OAC treatment-naïve biopsies. OCR and ECAR were measured in real-time using Seahorse Biosciences XFe24 analyser. (**A**) Percentage change from baseline of OCR following treatment with 0.1% DMSO control, 10 µM Pyrazinib (P3) or 6 µM Oligomycin for 24 h, (n = 10), Wilcoxon paired t-test, *p < 0.05. (**B**) Percentage change from baseline of ECAR following treatment with 0.1% DMSO control, 10 µM Pyrazinib (P3) or 6 µM Oligomycin for 24 h, (n = 10), Wilcoxon paired t-test. Data are expressed as + SEM.

Patient characteristics of the patient cohort evaluated in this study are outlined in Supplemental Table 1. Having demonstrated in real-time that OAC treatment-naïve biopsies are metabolically active we sought to investigate the effect of our anti-metabolic agent, pyrazinib (P3), on real-time metabolic rates in OAC treatment-naïve biopsies. Pyrazinib (P3) treatment significantly inhibited OCR in OAC treatment-naïve biopsies (*p* = 0.0039) (Fig. 2A). Pyrazinib (P3) induced a 35% reduction in OCR compared to the baseline OCR reading. Oligomycin, an ATP synthase inhibitor, was used as a positive control and resulted in a significant reduction in OCR (*p* = 0.0098) (Fig. 2A). No significant change in metabolic rate was seen following treatment with the 0.1% dimethyl sulfoxide (DMSO) control from baseline OCR (Fig. 2A). To determine if the reduction in OCR following treatment with pyrazinib (P3) was dependant on certain clinical patient characteristics, the percentage reduction in OCR was assessed according to the following clinical patient characteristics: nodal status, tumour stage, stage of differentiation and body mass index (Supplemental Fig. 3A,C,E,G). Importantly, the percentage reduction in OCR was independent of clinical patient characteristics suggesting that pyrazinib (P3) could function across our patient cohort. Regarding ECAR, treatment with pyrazinib (P3) did not significantly alter ECAR in OAC treatment-naïve biopsies (Fig. 2B). Percentage change in ECAR following treatment with pyrazinib (P3) was independent of patient characteristics: nodal status, tumour stage, stage of differentiation and body mass index (Supplemental Fig. 3B,D,F,H). Therefore, pyrazinib (P3) produced a significant reduction of oxygen consumption rate in OAC treatment-naïve biopsies and its function was independent of clinical patient characteristics.

### Pyrazinib (P3) significantly inhibited real-time metabolic rate in OAC treatment-naïve biopsies cultured under hypoxia (0.5% O_2_)

**Figure 3:**
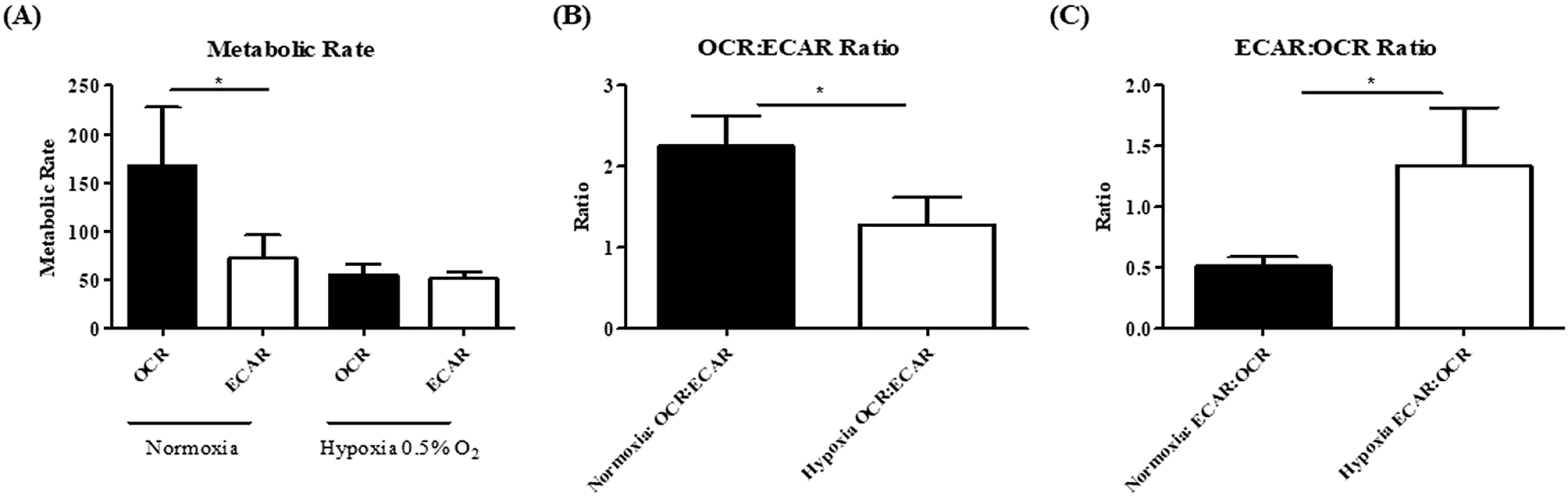
OAC treatment-naïve patient biopsies have an altered metabolic phenotype under hypoxic conditions (0.5% O_2_). OCR and ECAR were measured in real-time in male OAC treatment biopsies using Seahorse Technology. (**A**) Basal OCR and ECAR at baseline and post 6 h incubation under hypoxia 0.5% O_2_, (n = 7). (**B**) OCR:ECAR ratio of OAC pre-treatment biopsies cultured under normoxic and then hypoxic conditions, (n = 7). (**C**) ECAR:OCR ratio of OAC treatment-naïve biopsies cultured under normoxic and then hypoxic conditions, (n = 7). Wilcoxon signed rank t-test, *p < 0.05. Data expressed as + SEM.

**Figure 4:**
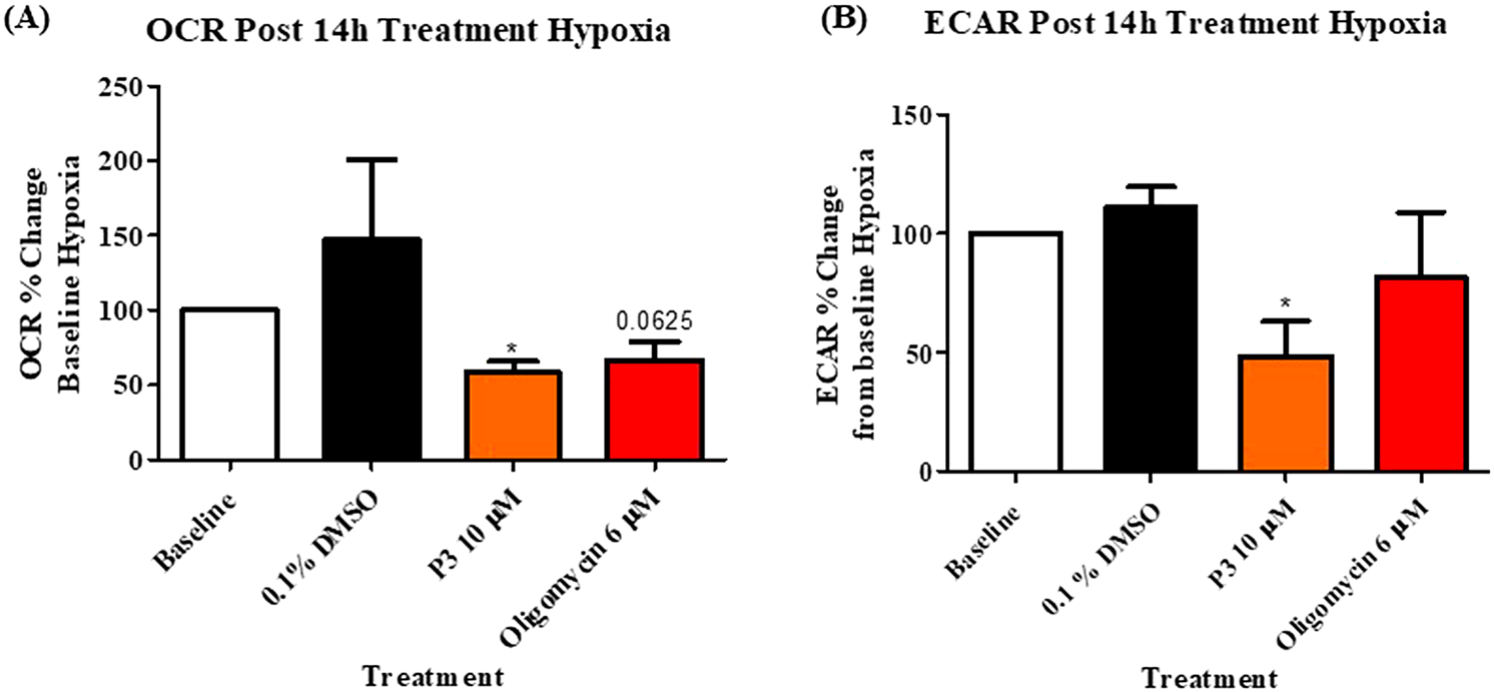
Pyrazinib (P3) significantly inhibits OCR and ECAR in OAC patient biopsies cultured under hypoxia (0.5% O_2_). (**A**) Percentage change from baseline OCR metabolic rate under hypoxia (0.5% O_2_) to OCR following treatment with 0.1% DMSO control, 10 µM Pyrazinib (P3) or 6 µM Oligomycin under 0.5% O_2_, (n = 6). (**B**) Percentage change from baseline of ECAR metabolic rate under hypoxia to ECAR following treatment with 0.1% DMSO control, 10 µM Pyrazinib (P3) or 6 µM Oligomycin under 0.5% O_2_, (n = 6). Wilcoxon signed rank t-test,*p < 0.05. Data expressed as mean + SEM.

Clinical patient characteristics of the patient cohort used in this study are outlined in Supplemental Table 2. Hypoxic tumours are inherently resistant to treatment thus we investigated both real-time metabolic rate and the action of pyrazinib (P3) under hypoxic conditions of 0.5% O_2_ in OAC treatment-naïve patient biopsies from 7 male patients. To determine if OAC biopsies adapt their metabolic rate to changes in oxygen levels, we evaluated real-time basal metabolic rate under normoxia and real-time metabolic rate of the same biopsies again following culture in 0.5% O_2_ for 6 h. Basal metabolic rate demonstrates OCR is significantly higher than ECAR under normoxic conditions (*p* = 0.0156) (Fig. 3A). In contrast to OAC biopsies cultured under normoxic conditions, following culture of OAC treatment-naïve biopsies under 0.5% O_2_, no significant differences were observed between OCR and ECAR (Fig. 3A). The ratio of OCR:ECAR was significantly higher in OAC treatment-naïve biopsies at baseline under normoxic conditions compared to the same biopsies cultured under hypoxia for 6 h (*p* = 0.0469) (Fig. 3B). Interestingly, the shift in metabolic phenotype in conditions of different oxygen concentrations highlights the ability of the OAC treatment-naïve biopsies to adapt to their local microenvironment, whereby there is a shift from oxidative phosphorylation to glycolysis under hypoxic conditions. Pyrazinib (P3) significantly inhibited OCR under hypoxia, resulting in a 41% reduction in real-time OCR (*p* = 0.0313) (Fig. 4A). Additionally, pyrazinib (P3) simultaneously and significantly inhibited real-time ECAR, a measure of glycolysis under hypoxia (*p* = 0.0313) (Fig. 4B). Pyrazinib (P3) induced a ~ 51% reduction in ECAR under hypoxia. In summary, our studies demonstrate the ability of OAC patient biopsies from male patients to dynamically adapt their metabolic rate in real-time to cope with changes in oxygen concentration in their local environment. Additionally, pyrazinib (P3) can significantly reduce OCR and ECAR under hypoxic conditions ex vivo in OAC treatment-naïve patient biopsies.

### OAC treatment-naïve biopsies display a heterogeneous inflammatory secretome

**Figure 5:**
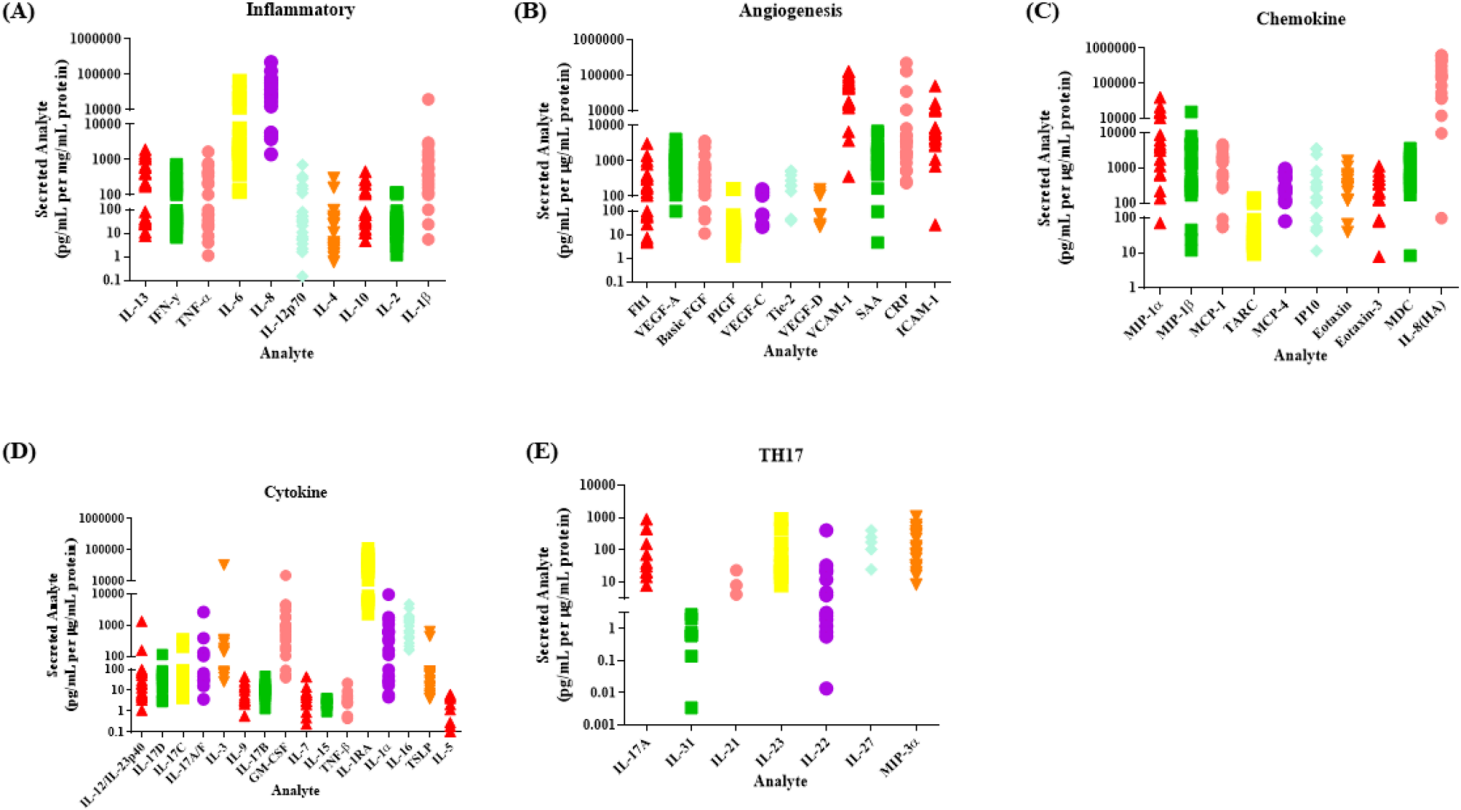
Secreted levels of 54 proteins from n = 22 OAC treatment-naïve biopsies cultured for 24 h were evaluated by multiplex ELISA. (**A**) Secreted levels of inflammatory proteins IL-13, IFN-γ, TNF-α, IL-6, IL-8, IL-12p70, IL-4, IL-10, IL-2 and IL-1β from OAC treatment-naïve biopsies. (**B**) Secreted levels of angiogenic and vascular injury proteins Flt1, VEGF-A, BasicFGF, PIGF, VEGF-C, TIE-2, VEGF-D, VCAM, SAA, CRP and ICAM from OAC treatment-naïve biopsies. (**C**) Secreted levels of chemokine proteins MIP-1α, MIP-1β, MCP, TARC, MCP-4 IP10, Eotaxin, Eotaxin-3, MDC, and IL-8(HA) from OAC treatment-naïve biopsies. (**D**) Secreted levels of cytokine proteins IL-12/IL-23p40, IL-17D, IL-17C, IL-17-A/F, IL-3, IL-9, IL-17B, GM-CSF, IL-7, IL-15, TNF-β, IL-1RA, IL-1α, IL-15, TSLP and IL-5 from OAC treatment-naïve biopsies. (**E**) Secreted levels TH17 pathway proteins IL-17A, IL-31, IL-21, IL-23, IL-22, IL-27 and MIP-3α from OAC treatment-naïve biopsies, (n = 22). Data normalised to protein content.

The clinical patient characteristics for the patient cohort used in this study are outlined in Supplemental Table 3. In addition to an altered metabolic phenotype, inflammation has been reported to play a significant role in the progression and treatment response of OAC tumours, whereby elevated levels of pro-inflammatory mediators such as LIF, C3a, C4a and IL-1β have been associated with poor treatment response^8,9,11^. To profile the inflammatory secretions of OAC treatment-naïve tumours, OAC biopsies were cultured for 24 h and the secreted levels of 54 proteins in the tumour conditioned media (TCM) were evaluated by multiplex ELISA. The secreted levels of 54 proteins were separated into 7 different panels including inflammatory, angiogenesis and vascular injury, chemokine, cytokine and T helper (T_H_)17 proteins. Secreted levels of selected inflammatory proteins IL-13, IFN-γ, TNF-α, IL-6, IL-8, IL-12p70, IL-4, IL-10, IL-2 and IL-1β from OAC treatment-naïve biopsies are shown in Fig. 5A. Secreted levels of angiogenic and vascular injury proteins Flt-1, VEGF-A, b-FGF, PIGF, VEGF-C, TIE-2, VEGF-D, VCAM, SAA, CRP and ICAM from OAC treatment-naïve biopsies are shown in Fig. 5B. Chemokine protein secretion including macrophage inflammatory protein (MIP)-1α, MIP-1β, MCP, TARC, MCP-4, IP-10, Eotaxin, Eotaxin-3, MDC, and IL-8(high sensitivity) from OAC treatment-naïve biopsies are illustrated in Fig. 5C. Secreted levels of cytokines IL-12/IL-23p40, IL-17D, IL-17A, IL-17C, IL-17-A/F, IL-23,IL-3, IL-9, IL-17B, GM-CSF, IL-7, IL-15, TNFβ, IL-1RA, IL-1α, IL-15, TSLP and IL-5 from OAC treatment-naïve biopsies are shown in Fig. 5D. T_H_17 pathway protein secretions of IL-17, IL-31, IL-21, IL23, IL-22, IL-27 and MIP-3α from OAC treatment-naïve biopsies are shown in Fig. 5E. Notably, there is a high level of variability of the secreted levels of the proteins detected in this screen, highlighting the high level of heterogeneity between OAC treatment-naïve patient tumours. Furthermore, no significant correlations were seen when secreted inflammatory factors were divided according to clinical patient characteristics including tumour stage, nodal status, body mass index, stage of differentiation and age at diagnosis (data not shown).

### Real-time metabolic profiles were significantly correlated with inflammatory secretions in OAC tumour biopsies

To investigate the relationship between real-time metabolic rate (OCR and ECAR) and the inflammatory protein secretions in OAC treatment-naïve biopsies, we correlated basal metabolic rate with inflammatory secretions in 10 matched patients, as per patient characteristics outlined in Supplemental Table 1.

OCR was significantly positively correlated with ECAR, (r = 0.8505, *p* < 0.0001). In addition, OCR was significantly correlated with the secreted levels of 3 of 54 proteins in the TCM including Vascular endothelial growth factor A (VEGF-A) (r = 0.7091, *p* = 0.0268), interleukin-1 receptor antagonist (IL-1RA) (r = 0.7939, *p* = 0.0088) and TSLP (r = 0.6727, *p* = 0.0390) shown in Table 1. ECAR was significantly positively correlated with OCR (r = 0.8505, *p* < 0.0001) and the secreted levels of 6 of 54 proteins in the TCM including VEGF-A (r = 0.8303, *p* = 0.0047), IL-1RA (r = 0.7455, *p* = 0.0174), TSLP (r = 0.7697, *p* = 0.0126), IL-13 (r = 0.7212, *p* = 0.0234), TNF-α (r = 0.6606, *p* = 0.0438) and macrophage inflammatory protein 3 alpha (MIP-3α) (r = 0.6606, *p* = 0.0438) shown in Table 2. Of note, Spearman correlations values of 0.4–0.59 are considered moderate, 0.6–0.79 are considered strong and 0.8–1.0 are considered very strong. Therefore, inflammatory protein secretions are significantly positively correlated with the real-time measures of metabolic rate; OCR and ECAR.

**Table 1:** Correlation of Metabolic rate with protein secretions in matched OAC treatment-naïve biopsies.

Correlation with oxygen consumption rate				
Factor	R value	P value	Significance	N
ECAR	0.8505	< 0.0001	***	17
VEGF-A	0.7091	0.0268	*	10
IL-1RA	0.7939	0.0088	**	10
TSLP	0.6727	0.0390	*	10

The protein secretions of 54 mediators analysed by multiplex ELISA were correlated with real-time metabolic rate readouts of OCR of matched patient biopsies. (**A**) OCR was significantly correlated with the rate of ECAR and the secreted levels of 3 of 54 proteins including VEGF-A, IL-1RA and TSLP in matched OAC treatment-naïve biopsies. Secretions from explant tissue and metabolic rate were normalised to protein content. Spearman correlations; Spearman r 0.4–0.59 moderate, 0.6–0.79 strong, 0.8–1.0 very strong; *p < 0.05, **p < 0.01, ***p < 0.0001.

**Table 2:** Correlation of metabolic rate with protein secretions in matched OAC treatment-naïve biopsies.

Correlation with extracellular acidification rate				
Factor	R value	P value	Significance	N
OCR	0.8505	< 0.0001	***	17
VEGF-A	0.8303	0.0047	**	10
IL-1RA	0.7455	0.0174	*	10
MIP-3α	0.6606	0.0438	*	10
TNF-α	0.6606	0.0438	*	10
IL-13	0.7212	0.0234	*	10
TSLP	0.7697	0.0126	*	10

The protein secretions of 54 mediators analysed by multiplex ELISA were correlated with real-time metabolic rate readouts of ECAR of matched patient biopsies. ECAR, a measure of glycolysis, was significantly correlated with the rate of OCR and the secreted levels of 6 of 54 proteins including VEGF-A, IL-1RA, MIP-3α, TNF-α, IL-13 and TSLP in matched OAC treatment-naïve biopsies. Secretions from explant tissue and metabolic rate were normalised to protein content. Spearman correlations; Spearman r 0.4–0.59 moderate, 0.6–0.79 strong, 0.8–1.0 very strong; *p < 0.05, **p < 0.01, ***p < 0.0001.

### Pyrazinib (P3) significantly altered the secretion of IL-1β, IL-3 and IL-17B from OAC treatment-naïve biopsies

**Figure 6:**
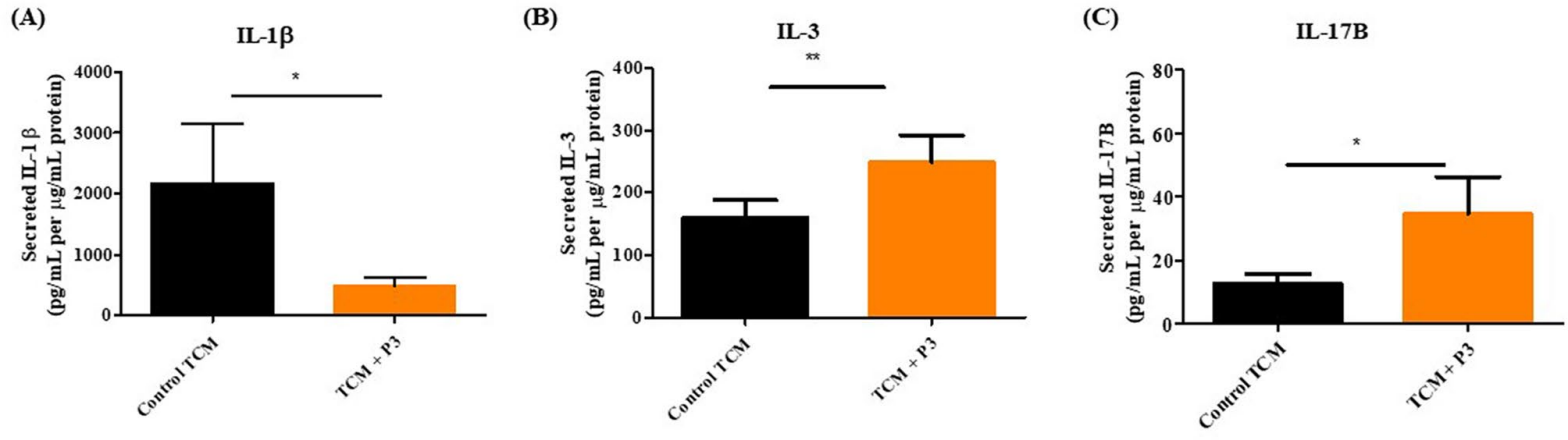
Pyrazinib (P3) significantly reduces secretion of IL-1β and increases secretion of IL-3 and IL-17B from OAC treatment biopsies. The effect of 24 h treatment of OAC treatment-naïve biopsies with 0.1% DMSO or 10 µM Pyrazinib (P3) on protein secretion was assessed by multiplex ELISA. (**A**) Pyrazinib (P3) significantly reduced the secretion of IL-1β from OAC treatment-naïve biopsies, (n = 22). (**B**) Pyrazinib (P3) significantly increases the secretion of IL-3 from OAC pre-treatment biopsies, (n = 15). (**C**) Pyrazinib (P3) significantly increases the secretion of IL-17B from OAC pre-treatment biopsies, (n = 15). Wilcoxon signed rank t-test,*p < 0.05, **p < 0.01. All data normalised to protein content. Data expressed as + SEM.

Inflammation drives development of OAC, however not all types of inflammation are detrimental to the host, *e.g.* T helper 1 (T_H_1) profiles are associated with a good response to immunotherapeutic drugs^14^, whereas myeloid cell abundance in tumours is associated with worse survival^15^. To investigate if our anti-metabolic pyrazine compound pyrazinib (P3) affects altered inflammatory secretions ex vivo, we cultured OAC treatment-naïve biopsies with 10 µM pyrazinib (P3) or 0.1% DMSO for 24 h and compared the inflammatory secretions from the OAC treatment-naïve biopsies from the same patient. Of the 54 factors screened for in the multiplex ELISA, pyrazinib (P3) treatment significantly alters the secretions of 3 proteins: IL-1β, IL-3 and IL-17B. Treatment with pyrazinib (P3) significantly reduced the secretion of IL-1β from OAC treatment-naïve tumour biopsies (*p* = 0.0377) (Fig. 6A). In contrast, pyrazinib (P3) significantly increased the secretions of IL-3 (*p* = 0.0020) and IL-17B (*p* = 0.0181) in ex vivo OAC treatment-naïve biopsies (Fig. 6B,C).

### Pyrazinib (P3) does not directly alter the expression of maturation markers on CD11c^+^ dendritic cells

**Figure 7:**
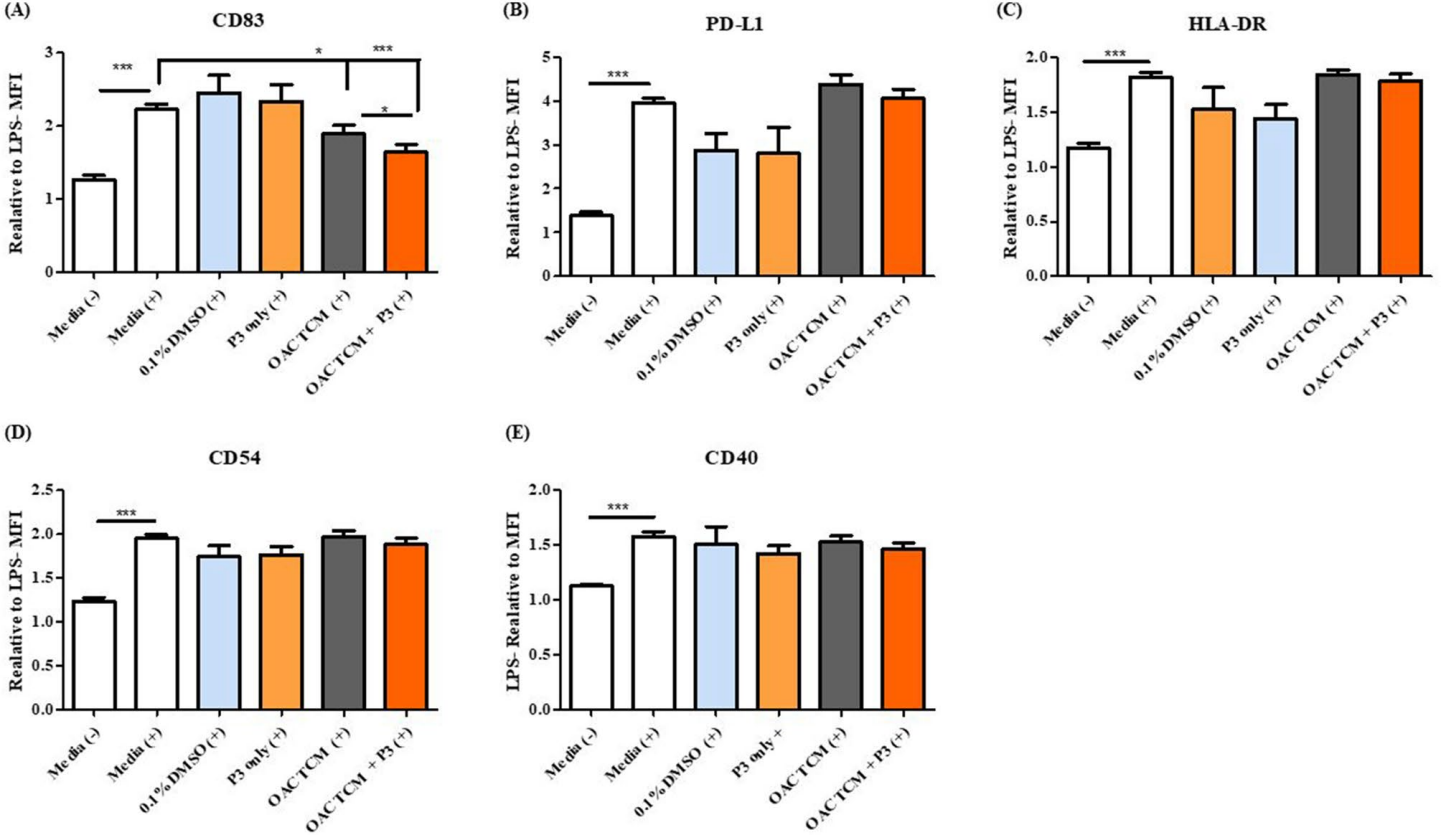
OAC TCM and Pyrazinib (P3) treated TCM significantly reduced expression of CD83 but did not alter the expression of PD-L1, CD40, HLA-DR or CD54 expression on monocyte derived dendritic cells following stimulation with LPS. The effect Pyrazinib (P3) and Pyrazinib (P3) treated TCM on the expression of dendritic cells maturations markers was assessed by flow cytometry. (**A**) CD83 expression on monocyte derived dendritic cells following incubation with LPS- and LPS + M199, 0.1% DMSO + LPS, 10 µM Pyrazinib (P3) + LPS, 0.1% DMSO treated OAC TCM + LPS and OAC TCM treated with Pyrazinib (P3) + LPS. (**B**) PD-L1 expression on monocyte derived dendritic cells following incubation with LPS- and LPS + M199, 0.1% DMSO + LPS, 10 µM Pyrazinib (P3) + LPS, 0.1% DMSO treated OAC TCM + LPS and OAC TCM treated with 10 µM Pyrazinib (P3) + LPS. (**C**) HLA-DR expression on monocyte derived dendritic cells following incubation with LPS- and LPS + M199, 0.1% DMSO + LPS, 10 µM Pyrazinib (P3) + LPS, 0.1% DMSO treated OAC TCM + LPS and OAC TCM treated with 10 µM Pyrazinib (P3) + LPS. (**D**) CD54 expression on monocyte derived dendritic cells following incubation with LPS- and LPS + M199, 0.1% DMSO + LPS, 10 µM Pyrazinib (P3) + LPS, 0.1% DMSO treated OAC TCM + LPS and OAC TCM treated with 10 µM P3 + LPS. (**E**) CD40 expression on monocyte derived dendritic cells following incubation with LPS- and LPS + M199, 0.1% DMSO + LPS, 10 µM Pyrazinib (P3) + LPS, 0.1 DMSO treated OAC TCM + LPS and OAC TCM treated with 10 µM Pyrazinib (P3) + LPS. LPS stimulation indicated by (+). Wilcoxon signed rank t-test, *p < 0.05,***p < 0.001. Data expressed as + SEM.

In addition to the anti-metabolic and anti-inflammatory activity of pyrazinib (P3), it was critical to investigate its effect on immune cells, including dendritic cells which play a critical role in anti-tumoural immunity. Thus, we investigated whether pyrazinib (P3) altered the expression of dendritic cell maturation markers. Dendritic cells are professional antigen presenting cells which play a key role in orchestrating anti-tumour immune responses, via T cell polarisation and activation. In the development of a new compound, it is crucial to determine if such treatment will affect the function of important anti-tumour immune cells such as dendritic cells, as this could hinder clinical potential. Patient information is outlined in Supplemental Table 3. Monocyte-derived immature dendritic cells were pre-incubated with either 0.1% DMSO, 10 µM pyrazinib (P3), 0.1% DMSO treated tumour conditioned media (TCM) or 10 µM pyrazinib (P3)-treated TCM for 24 h before LPS was added to induce dendritic cell maturation. To assess the effect of the following treatments; vehicle control 0.1% DMSO, pyrazinib (P3), vehicle control treated TCM and TCM pre-treated with pyrazinib (P3) on the expression of dendritic cell maturation markers, the levels of CD83, PD-L1, CD40, HLA-DR and CD54 on CD11c^+^ cells was assessed by flow cytometry**.** Direct treatment of dendritic cells with either 0.1% DMSO or 10 µM pyrazinib (P3) following LPS stimulation did not significantly alter the expression of CD83, PD-L1, CD40, HLA-DR and CD54 dendritic cell maturation markers (Fig. 7A–E). Pyrazinib (P3) treated TCM significantly (*p* = 0.0004) reduced expression of CD83 on dendritic cells (Fig. 7A) and resulted in a 15% greater reduction in CD83 expression on dendritic cells compared to control treated TCM (*p* = 0.0100). A representative histogram is shown in Supplemental Fig. 4. No significant differences in PD-L1, CD40, HLA-DR and CD54 expression on dendritic cells were seen following treatment with either control or pyrazinib (P3)-treated TCM (Fig. 7B–E). In summary, pyrazinib (P3) does not directly affect the expression of dendritic cell maturation makers, PD-L1, CD40, HLA-DR and CD54. Secreted factors from OAC tumour biopsies can significantly reduce CD83 expression on dendritic cells but not the expression of PD-L1, CD40, HLA-DR and CD54.

### Pyrazinib (P3) does not alter T cell viability in vitro

**Figure 8:**
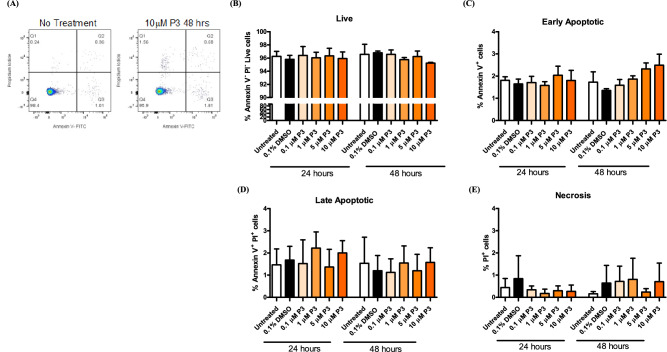
Pyrazinib (P3) does not alter T cell viability. The effect of Pyrazinib (P3) on Jurkat cell viability was assessed by flow cytometry. (**A**) Representive flow plot of utreated and Pyrazinib (P3) treated jurkat cells. The percentage of (**B**) live (**C**) early apoptotic (**D**) late apoptotic and (**E**) necrotic jurkat cells following 24 or 48 h treatment with media only, 0.1% DMSO control of 0.1, 1, 5 or 10 µM of Pyrazinib (P3), (n = 3). Paired t-tests.

To determine if treatment with pyrazinib (P3) was toxic to T cells, the Jurkat T cell line was treated with varying doses of pyrazinib (P3) at 0.1, 1, 5 & 10 μM), for 24 and 48 h, and stained with FITC-conjugated Annexin V and PI and analysed by flow cytometry. Treatment of Jurkat cells with increasing concentrations of pyrazinib (P3) did not significantly affect Jurkat cell viability, at either time point tested, compared to 0.1% DMSO control, with respect to the percentage of live (Fig. 8A), early apoptotic (Fig. 8B), late apoptotic (Fig. 8C) and necrotic cells (Fig. 8D). The percentage of live cells treated with the varying concentrations of pyrazinib (P3) at 24 h and 48 h incubation periods did not differ, with a maintained range of 94–98% Jurkat cell viability. There was some variance seen in the proportion of cells that underwent early apoptosis (Annexin V + only), but at very low levels (≤ 3%), therefore no significant difference in the percentage of cells that underwent early apoptosis. The percentage of cells that underwent necrosis (PI^+^ only) remained at very low levels (≤ 3%) with no significant difference observed between samples. Late apoptosis (Annexin V^+^, PI^+^) was detected in ≤ 2% of cells, and there was no significant difference between different treatment conditions. In summary, pyrazinib (P3) is not toxic to Jurkat T cells, at 0–10 μM concentrations after 24 h and 48 h treatment.

### Pyrazinib (P3) does not significantly alter the expression of T cell activation markers in vitro

**Figure 9:**
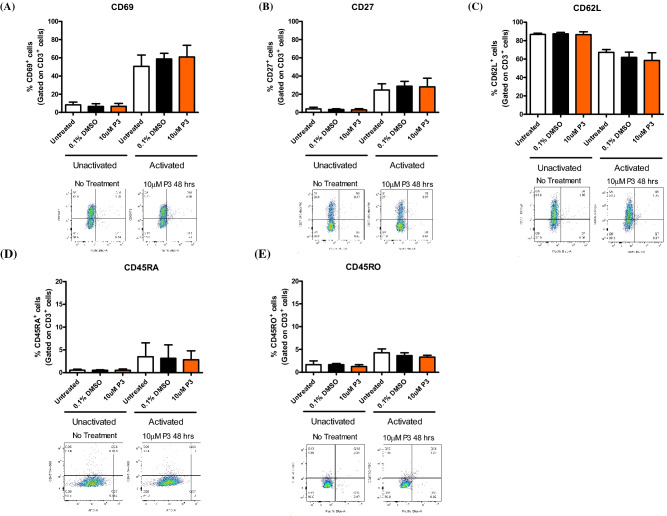
Pyrazinib (P3) does not significantly alter T cell activation marker expression. The effect of Pyrazinib (P3) on Jurkat cell activation marker expression was assessed by flow cytometry. The expression of (**A**) CD69, (**B**) CD27, (**C**) CD62L, (**D**) CD45RA and (**E**) CD45RO on Jurkat cells following treatment with media only, 0.1% DMSO control or 10 µM Pyrazinib (P3), (n = 3). Jurkat cells were activated with anti-CD3/CD28 antibodies. Statistical analysis was carried out using paired t-tests.

To further investigate the effect of pyrazinib (P3) on the immune system, we investigated the effect of pyrazinib (P3) treatment on the expression levels of the T cell activation markers CD27, CD62L, CD69, CD45RO and CD45RA on Jurkat cells. To investigate if treatment with pyrazinib (P3) altered activation status in both activated and unactivated T cells, cell surface expression of a range of activation markers was assessed by flow cytometry. There was no difference in samples treated with 10 µM pyrazinib compared to control treated cells, in respect to the percentage of cells expressing CD27, CD62L, CD69, CD45RO and CD45RA antibodies (Fig. 9A–E). It can be concluded that changes in T cell marker expression are minimal in T cells treated with 10 μM pyrazinib for 24 h compared to vehicle control samples, in both activated and unactivated Jurkat cells.

## Discussion

This study highlights a novel method for measuring the real-time metabolic profiles of OAC treatment-naïve tumour biopsies which could be applied to multiple cancer types.

Ex vivo, real-time metabolic profiling demonstrated that oxidative phosphorylation was significantly higher in OAC treatment-naïve biopsies compared to glycolysis. This supports previous findings by our group which have reported the importance of oxidative phosphorylation in OAC, and its previous association with radiation resistance^10^. The metabolic rate of OAC biopsies was shown to be independent of clinical patient characteristics. Whilst Warburg initially found cancer cells to be reliant on aerobic glycolysis, numerous studies also support the functional role of oxidative phosphorylation in tumorigenesis^1^. Real-time metabolic profiling of OAC biopsies supports previous findings at the in vitro level, which demonstrates that mitochondrial respiration is a predominant metabolic pathway used by OAC cancer cells^10^. Numerous studies have evaluated the mitochondrial function of tumour cells and reported that tumour cells predominantly have functional mitochondria which have retained the ability to carry out oxidative phosphorylation^16^.

Our novel small molecule compound pyrazinib (P3) significantly inhibited OCR in OAC treatment-naïve biopsies and no significant upregulation of ECAR occurred following treatment with pyrazinib (P3). Importantly, the activity of pyrazinib (P3) was independent of clinical patient characteristics, indicating that pyrazinib (P3) can maintain its anti-metabolic activity irrespective of patient’s clinical characteristics such as tumour stage, nodal status, tumour differentiation or patient BMI. The significant effect of pyrazinib (P3) on OAC tumour oxidative phosphorylation rate is not only likely to have an anti-cancer effect given the prominent utility of oxidative phosphorylation in OAC tumours, it may also enhance the radioresponse of these tissues, as seen previously in vitro in an isogenic model of OAC radio-resistance^7^. Targeting oxidative phosphorylation has been reported in a number of studies as a novel mechanism to enhance radiosensitivity and reduce tumour growth^5,17^. A study by Benej et al. demonstrated that targeting mitochondrial respiration with the ergot alkaloid papaverine significantly inhibited mitochondrial respiration and enhanced tumour oxygenation and subsequently enhanced radiosensitivity in pulmonary adenocarcinoma cells^17^. An interesting study carried out by Bol et al. also demonstrated reprogramming of tumour mitochondria improves responses to radiation, a mitochondrial dysfunctional cell line which were exclusively glycolytic were found to be more radiosensitive than wild type oxidative phosphorylation proficient cells^18^. A novel in vivo model of oncogenic ablation-resistant pancreatic cancer cells which were responsible for tumour relapse were reported to depend on mitochondrial function for survival^5^. Targeting oxidative phosphorylation in this subpopulation of surviving pancreatic cells was shown to significantly decrease tumour spheroid growth^5^. Furthermore, oxidative phosphorylation is significantly upregulated in breast cancers deficient in RB1, a protein lost in 20–30% of basal-like breast cancers^19,20^. Tigecycline, a mitochondrial translational inhibitor attenuated growth of RB1-deficient breast tumours in vivo^20^. Taken together with the findings in this study, targeting oxidative phosphorylation in the neo-adjuvant setting could enhance radiosensitivity in OAC, but also oxidative phosphorylation could be targeted in the adjuvant setting to specifically target any remaining surviving cancer cells. Given the predominance of the oxidative phosphorylation pathway in OAC tumours, such a therapeutic strategy strongly warrants further studies. It would be of interest in the future to further validate our findings in a larger patient cohort to eliminate any clinical individual differences. Additionally, several metabolomic based studies have evaluated the importance of energy metabolism and metabolic reprogramming in oesophageal cancer. A study by Zhang et al. identified 8 metabolites which were significantly higher in the serum of OAC patients compared to healthy controls. Importantly, of the significantly elevated metabolites, citrate, lactate and glucose which function in oxidative phosphorylation, glycolysis and gluconeogenesis respectively were all significantly upregulated in OAC patients highlighting the altered metabolic profile of OAC patient serum compared to healthy controls^21^. A study by Tokunaga et al. demonstrated significant metabolic alterations between paired resected oesophageal SCC tissue and non-tumour oesophageal squamous cell carcinoma (SCC) tissue samples^22^. Interestingly, this study found the levels of both malic acid and citric acid were significantly lower in more advanced SCC tumours when compared to early stage tumours which may be associated with a downregulation of the tricarboxylic cycle in late stage tumours^22^. Furthermore, a metabolomics study which utilised urinary samples from SCC patients and healthy controls demonstrated oesophageal SCC was associated with alterations in fatty acid β-oxidation and the metabolism of purines, amino acids, and pyrimidines^23^. These studies amongst others highlight the importance of metabolic alterations in oesophageal cancer compared to healthy controls and highlight the potential for biomarker development for disease diagnosis and progression^24,25^. It would be of interest to employ a similar approach in OAC treatment-naïve tumour tissue across the various stages of disease progression to further elucidate the role of altered energy metabolism in OAC and compare the findings to real-time metabolic analysis.

Hypoxia promotes the transformation of tumour cell metabolism from oxidative metabolism to anaerobic glycolysis, which protects tumour cells, promotes tumour growth and the development of treatment resistance in tumour stem cells^26^. We sought to investigate if OAC treatment-naïve biopsies adapt their metabolic profile to conditions of hypoxia and if pyrazinib (P3) could inhibit mitochondrial respiration under hypoxic conditions of 0.5% O_2_. Under normoxic conditions, OAC tumours had a significantly higher rate of OCR, but adapted their metabolic rate to cope with hypoxic conditions, therefore there was no significant difference in OCR compared to ECAR. The OCR:ECAR ratio was significantly higher in OAC tumour under normoxia versus hypoxia and the ECAR:OCR ratio was significantly higher in hypoxic versus normoxic biopsies, highlighting the metabolic adaptation of OAC tumours to their environment. Pyrazinib (P3) significantly inhibited both oxidative phosphorylation and glycolysis. The ability of pyrazinib (P3) to inhibit both oxidative phosphorylation and glycolysis is a critical finding, which shows even in OAC tumours which can adapt their metabolic profiles to hypoxic conditions, pyrazinib (P3) can still inhibit both OCR and ECAR. In a study by Wang et al., in genetically modified macrophages overexpressing HIF-1α the OCR:ECAR ratio was dramatically decreased compared to non-HIF-1α overexpressing macrophages demonstrating a shift to glycolysis metabolism compared to mitochondrial oxidation in HIF-1α overexpressing macrophages^27^. Oxygen is a potent radiosensitiser and solid tumours with areas of hypoxia are the most aggressive and difficult tumours to treat^26^. A number of strategies which have attempted to increase oxygen delivery to the tumour have failed in the clinic largely due to the heterogenic nature of tumour vasculature^28^. In a pancreatic xenograft, the selective HIF-1α inhibitor PX-478 was found to potentiate the effect of fractioned chemoradiation therapy^29^. Targeting intra-tumoural oxygen consumption with compounds targeting oxidative phosphorylation may present a novel means to overcome tumour hypoxia and enhance anti-cancer activity in tumours where oxidative phosphorylation is upregulated, but also to improve treatment response rates in hypoxic treatment resistant tumours^17^. Notably, elevation of oxygen by as little as 2% is sufficient to produce oxygen enhancement^16^. Targeting oxidative phosphorylation in mammary tumours with papaverine was found to enhance hypoxic tumour oxygenation, sensitise tumours to radiation therapy and significantly reduce tumour growth^17^. Taken together with our current and our previous findings in vitro which demonstrated the anti-metabolic and radiosensitising activity of pyrazinib (P3) suggests pyrazinib (P3) has the potential to function as an anti-cancer agent in vivo^7^. Of note, the fresh patient samples used in our hypoxia metabolism study were all male, whilst OAC is a male dominant disease, previous studies have suggested a gender bias may exist in oesophageal cancer patients in relation to treatment response, thus it would be of importance to address the influence of gender on hypoxia metabolism in a much larger prospective study across multiple sites^30^.

Tumour metabolism is tightly linked with both the local and systemic inflammatory response. OAC is an inflammatory driven upper gastrointestinal cancer^11^, thus we sought to characterise the inflammatory profile of the tumour conditioned media from OAC treatment-naïve biopsies. A multiplex ELISA demonstrated the heterogeneity of secreted factors from OAC biopsies including inflammatory, angiogenic and vascular injury, chemokine, cytokine and T_H_17 related proteins. To investigate a potential relationship between metabolic rate and the OAC inflammatory secretion profile, we correlated protein secretions with baseline real-time metabolic rate in matched patient samples. OCR was significantly correlated with ECAR in all patients at baseline. Both OCR and ECAR were significantly positively correlated with the secretion of VEGF-A, IL-1RA and TSLP in OAC treatment-naïve biopsies. In addition, ECAR was positively correlated with IL-13, MIP-3α and TNF-α. Oxidative phosphorylation and glycolysis are known to be influenced by systemic inflammation thus it is not surprising that metabolic rate correlated with a number of inflammatory mediators^31^. The significant correlation between VEGF-A secretion and OCR and ECAR highlights the tight links which exist between the two biological processes of angiogenesis and metabolism, whereby there are elevated levels of the angiogenic mediators VEGF-A and TSLP in tumours with higher levels of oxidative phosphorylation^32,33^. TNF-α was only significantly correlated with ECAR and not OCR in OAC treatment-naïve biopsies. TNF-α was previously shown to induce aerobic metabolism in prostate epithelial cells and glycolytic reliance in mammary carcinoma cells^34,35^. In addition, in a previous study, MIP-3α was shown to be significantly correlated with the levels of HIF-1α, a mediator of glycolytic induction, in Barrett’s oesophagus tissue^36^.

Treatment of OAC treatment-naïve biopsies with pyrazinib (P3) significantly inhibited IL-1β secretion and increased IL-3 and IL-17B secretion. The significant reduction of IL-1β secretion following pyrazinib (P3) treatment is a critical finding which may contribute to pyrazinib’s (P3) anti-cancer effect. Previous work by our department found elevated levels of IL-1β in tumour samples compared to squamous epithelium from the same patients, and IL-1β levels were significantly decreased in the TCM generated from post treatment biopsies, compared to the TCM generated from pre-treatment biopsies in matched patients who achieved a complete pathological response to neoCRT^9^. In addition, IL-1β was previously shown to be significantly correlated with clinical outcome in oesophageal SCC, whereby patients with IL-1β positive tumours had a poor response to treatment compared to patients with IL-1β negative tumours, the suggested underlying mechanism of this difference in tumour response was increased epithelial–mesenchymal transition aggressive tumour growth in IL-1β-positive tumours^37^. Inhibition of IL-1β was shown to attenuate tumour growth and invasion and ameliorate treatment resistance^37^. Furthermore, in a study by Deans et al., tumoural IL-1β expression levels were significantly correlated with systemic inflammation as measured by C-reactive proteins levels, which is a marker of reduced survival in oesophagogastric cancer patients^38^. In an in vivo melanoma model, IL-1β inhibition was shown to stably reduce tumour growth by limiting inflammation and inducing the maturation of immature myeloid cells into M1 macrophages. Furthermore, in an in vitro model of pancreatic chemoresistance, administration of an IL-1 receptor blocking antibody, as a means of targeting IL-1β signalling, reduced NF-κB activation and the acquisition of chemoresistance in these cells^39^. Reports from the literature suggest the significant inhibition of IL-1β in response to pyrazinib (P3) is a positive effect which may contribute to the anti-cancer activity of this drug in addition to its effects on oxidative phosphorylation in vitro and ex vivo and radiosensitivity in vitro. IL-3 has been reported to exert paradoxical effects in cancer including pro-tumourigenic as well as anti-tumourigenic cellular responses^40,41^. Importantly, IL-3 has been reported to play an important role in anti-tumoural immunity. IL-3 is able to enhance antigen presentation by dendritic cells and activate macrophages to increase the expression of class II MHC molecules and IL-1^42^. Elevated gene expression of IL-3 in fibrosarcoma xenografts (FSA-JmIL-3 tumours) was associated with enhanced response to radiation compared to parental tumours, where FSA-JmIL-3 tumours were associated with increased lymphocyte infiltration and elicited immune responses^41^, suggesting that the enhanced secretion of IL-3 following pyrazinib (P3) treatment is a positive effect of this small molecule compound.

Dendritic cells are professional antigen presenting cells which are responsible for induction of antigen specific T cell responses, thus it is critical that function of dendritic cells remains intact even in the presence of our small molecule compound pyrazinib (P3). In this study, we investigated both the effect of the secretions from the TCM and pyrazinib (P3) on the expression of dendritic cell maturation markers. Increased expression of several cell surface markers including CD54, PD-L1, CD40, CD83, and HLA-DR on dendritic cells is associated with dendritic cell maturation and T cell activating ability^43^. Direct treatment with pyrazinib (P3) showed no effect on CD83, CD54, PD-L1, CD40 and HLA-DR expression in response to LPS, whereas both control and pyrazinib (P3) treated TCM significantly reduced the expression of CD83, suggesting that mediators secreted from the tumour microenvironment specifically exert an inhibitory effect on dendritic cell maturation. Pyrazinib (P3) treatment does not negatively affect the expression of dendritic cell maturation markers, indicating the function of these cells remains intact even in presence of this compound. This is a critical finding, as a previous study by our group demonstrated that both control and bevacizumab treated colorectal conditioned media significantly inhibited LPS-induced maturation and function of dendritic cells^43^.

The adaptive immune system is associated with tumour control and elimination, particularly the T_H_1 phenotype^14,44,45^. Pyrazinib (P3) did not significantly alter the viability of a Jurkat T cell line. This is an important finding, which highlights the potential to use pyrazinib (P3) within the clinical setting because it does not deplete or kill T cells. We also examined the effect of pyrazinib on Jurkat T cell activation status, using pre-activated Jurkats. Pyrazinib (P3) has been previously shown to enhance radiosensitivity in vitro and a study by Voos et al. demonstrated that radiation doses of ≥ 2 Gy activate Jurkat T cells and stimulates pro-inflammatory immune responses, through upregulation of IL-2, IFN-γ and CD25 surface expression^46^. Importantly, pyrazinib (P3) did not affect expression of T cell activation markers by activated or unactivated Jurkat cells. CD8^+^ T cells are more susceptible to becoming exhausted upon constitutive activation than CD4^+^ T cells^47^ and one of the limitations to this study is the use of Jurkat T cells in this in vitro setting. Further research is required in relation to this study, both in patient-derived PBMCs and in the in vivo setting at multiple timepoints to gain a better understanding of the effect pyrazinib (P3) may have on other immune cells within the tumour microenvironment.

In summary, we report a new method for profiling the metabolic rate of human OAC tumour biopsies in real time, highlighting the importance of the oxidative phosphorylation pathway in OAC tumours, and that these tumours can adapt their metabolic profiles in line with changes in oxygen tension. We have demonstrated the novel anti-metabolic and anti-inflammatory action of pyrazinib (P3) in ex vivo OAC treatment-naïve biopsies, in addition to its radiosensitising properties. It will be critical to further evaluate the anti-cancer potential of pyrazinib (P3) now in a murine model of OAC.

## Methods

### Small molecule compound

Pyrazinib (P3), (E)-2-(2-Pyrazin-2-yl-vinyl)-phenol was synthesised by Onyx Scientific (UK). Pyrazinib (P3) was dissolved in 100% DMSO to make 10 mM stock solutions for experimental use and stored at − 20 °C.

### Patient samples

Following ethical approval (Joint St James’s Hospital/AMNCH ethical review board) and written informed consent, diagnostic biopsy specimens were taken from OAC patients being treated with curative intent, by a qualified endoscopist, prior to neo-adjuvant therapy. Histologic confirmation of tumour tissue in biopsies was performed by a pathologist using routine haematoxylin and eosin staining. All patient tumour tissue used in this study was taken prior to the initiation of neo-adjuvant treatment (treatment-naïve tissues). All experimental protocols were approved by the joint St James’s Hospital/AMNCH ethical review board and carried out in accordance with the relevant guidelines of the joint St James’s Hospital/AMNCH ethical review board.

### Real-time metabolic profiling of OAC tumour biopsies

Three biopsies per patient were collected at endoscopy, immediately placed on saline-soaked gauze and were transported to the laboratory within 10 min. Each biopsy was placed into a separate well of a 24 well XF24 Islet Capture Microplate (Agilent Technologies, Santa Clara, CA, USA) containing 1 mL M199 medium (Gibco) supplemented with 10% FBS (Gibco), 1 μg/mL insulin (Sigma) and 1% penicillin/streptomycin (Gibco). 1 mL of complete M199 was placed in four background control wells. A XF24 capture screen was placed over each biopsy to prevent the biopsy touching utility plate probes during assay. The XF24 microplate was placed at 37 °C for 30 min to allow biopsies to equilibrate. Three baseline measurements of OCR and ECAR were taken over 24 min consisting of three repeats of mix (3 min), wait (2 min), measurement (3 min) to establish basal respiration, using a Seahorse Biosciences XFe24 analyser (Agilent Technologies, Santa Clara, CA, USA). Basal respiration of each patient was established by taking the average OCR and ECAR readout from the three individual biopsies obtained from same patient. Following basal metabolic profiling of biopsies, capture screens were removed and biopsies and corresponding media were transferred to a new XF24 islet capture microplate and treated with one of the following; 0.1% DMSO (control), 6 µM of oligomycin (positive control) or 10 µM of pyrazinib (P3). Following treatment biopsies were cultured for 24 h at 37 °C in 5% CO_2_/95% air. Following 24 h culture, a capture screen was placed on each biopsy and three basal measurements of OCR and ECAR were taken over 24 min consisting of three repeats of mix (3 min), wait (2 min), measurement (3 min) to establish the effect of drug treatment with our novel small molecule pyrazinib (P3) on OCR and ECAR. The effect of treatment was determined as the percentage change in metabolic rate readout from the baseline reading of each individual biopsy to the reading following treatment of that individual biopsy. The metabolic rate of each biopsy was normalised to tumour protein content using the BCA assay (Pierce) and tumour biopsies were snap frozen and stored at − 80 °C. Tumour conditioned media (TCM) was collected and stored at − 80 °C.

### Real-time metabolic profiling of OAC tumour biopsies cultured under hypoxic conditions

Basal metabolic rate was determined as described as stated above. Following evaluation of basal OCR and ECAR using the Seahorse Biosciences XFe24 analyser; biopsies and corresponding media were transferred to a new XF24 islet capture microplate and cultured in the Whitley H35 hypoxystation (Don Whitley Scientific) at 0.5% O_2_ at 37 °C in 5% CO_2_ for 6 h. Following 6 h culture, capture screens were placed over each biopsy in each well and the plate was transferred to Whitley i2 workstation containing the XFe24 Seahorse analyser maintained at 0.5% O_2._ Real-time OCR and ECAR were assessed under 0.5% O_2_, three measurements of OCR and ECAR were taken over 24 min consisting of three repeats of mix (3 min), wait (2 min) and measurement (3 min), to establish the effect of a 6 h hypoxia culture on real-time metabolic rate in OAC patient biopsies. Following metabolic profiling of biopsies, capture screens were removed and biopsies and corresponding media were transferred to a new XF24 islet capture microplate and treated with one of the following; 0.1% DMSO (control), 6 µM of oligomycin (positive control) or 10 µM of pyrazinib (P3) for 14 h under 0.5% O_2_ at 37 °C in 5% CO_2._ Following 14 h treatment, real-time OCR and ECAR measurements were taken over 24 min consisting of three repeats of mix (3 min), wait (2 min), measurement (3 min) whilst the Seahorse Biosciences XFe24 analyser was maintained under 0.5% O_2_ to establish the effect of treatment on metabolic rate of OAC biopsies maintained under 0.5% O_2._ Following metabolic profiling, capture screens were removed, biopsies were snap frozen and both biopsies and TCM were stored at − 80 °C. The metabolic rate of each biopsy was normalised to tumour protein content using the BCA assay (Pierce).

### Multiplex ELISA quantification of 54 soluble tumour proteins

TCM, generated as above, was screened for the presence of tumour-secreted cytokines and angiogenic growth factors by multiplex ELISA (Meso Scale Diagnostics, USA). Angiogenic, vascular injury, inflammatory cytokine and chemokine secretions were measured using a 54-plex kit spread across 7 plates, used to quantify the secretions of CRP, Eotaxin, Eotaxin-3, FGF (basic), GM-CSF, ICAM-1, IFN-γ, IL-10, IL-12/IL-23p40, IL-12p70, IL-13, IL-15, IL-16, IL-17A, IL-17A/F, IL-17B, IL-17C, IL-17D, IL-1RA, IL-1α, IL-1β, IL-2, IL-21, IL-22, IL-23, IL-27, IL-3, IL-31, IL-4, IL-5, IL-6, IL-7, IL-8, IL-8 (high sensitivity), IL-9, IP-10, MCP-1, MCP-4, MDC, MIP-1α, MIP-1β, MIP-3α, PIGF, SAA, TARC, Tie-2, TNF-α, TNF-β, TSLP, VCAM-1, VEGF-A, VEGF-C, VEGF-D and VEGFR-1/Flt-1 from OAC TCM previously treated with 10 µM pyrazinib (P3) or vehicle control (0.1% DMSO) for 24 h. All samples were run neat, except for CRP, SAA, V-CAM and I-CAM, which were diluted 1:4. Angiogenesis and Vascular Injury plates were run in one day, as per manufacturer’s recommendations, while all other plates were run with an extended overnight sample incubation step at 4 °C (manufacturer’s Alternate Protocol 1). Data was analysed using MSD Discovery Workbench software version 4.0 to calculate protein concentration (pg/ml) from standard curves. Secretion data was normalised appropriately to total tumour protein content in the biopsies using the BCA assay (Pierce).

### Dendritic cell isolation and culture

Human monocyte-derived immature dendritic cells were generated from peripheral blood mononuclear cells (PBMCs) obtained from buffy coat preparations (National Blood Centre, St. James’s Hospital, Dublin) by density gradient centrifugation (Lymphoprep) as described^43,48^. Briefly, monocytes were isolated by positive selection using anti-CD14 magnetic microbeads, as described by the manufacturer (Miltenyi Biotec) and seeded at a density of 1 × 10^6^ cells/mL in 6-well plates in 3 mL of Roswell Park Memorial Institute (RPMI)-1640 medium (Gibco) containing 10% defined HyClone FBS (Thermo Scientific), 1% penicillin/streptomycin (Lonza, Basel, Switzerland), 1% fungizone (Sigma-Aldrich), human granulocyte macrophage colony-stimulating factor (GM-CSF, 50 ng/mL; Immunotools, Germany), and human IL-4 (70 ng/mL; Immunotools) in a humidified atmosphere with 5% CO_2_ at 37 °C. Cells were fed at day 3 by replacing half the medium with fresh cytokine-supplemented RPMI. At day 6, cells exhibited a CD11c^+^ immature dendritic cell phenotype.

### Stimulation of monocyte-derived dendritic cells

Six day old monocyte-derived dendritic cells were plated in 96-well plates at 2 × 10^5^ cells in 200 µL RPMI-1640 media supplemented with 10% defined Hyclone FBS (Thermo Scientific) and stimulated with a 1:2 dilution of control TCM or pyrazinib (P3) treated TCM from OAC tumour explants or 0.1% DMSO or 10 µM pyrazinib (P3) for 4–5 h before exposure to 10 µg/mL of ultra-pure TLR4 agonist *Escherichia coli* lipopolysaccharide (LPS-EB; Invivogen) overnight, incubating at 37 °C, 5% CO_2_. Supernatants were harvested and snap frozen, and cells were assessed for expression of surface markers.

### Flow cytometry

Dendritic cells were stained with the following antibodies: anti-CD83 Pe-Cy7, anti-PD-L1 Brilliant Violet 421, anti-CD11c Brilliant Violet 510, anti-CD54 allophycocyanin (APC), and anti-HLA-DR APC-Cy7 (Biolegend) for 15 min at room temperature in the dark. Samples were washed in PBA buffer (PBS containing 2% FCS and 0.01% v/v sodium azide) and acquired on DAKO CyAn flow cytometer (Beckman Coulter).Compensation was performed using positive and negative compensation beads (BD Biosciences) and data were analysed with FlowJo software (Tree Star Inc.) version 5^43,48^.

### Annexin V propidium iodide

Jurkat cells were seeded 5 × 104 cells/mL into 12 well plates in complete Roswell Park Memorial Institute (RPMI) 1,640 medium supplemented with 10% fetal calf serum (Lonza, Basal, Switzerland) and 1% penicillin–streptomycin (Lonza, Basal, Switzerland). Cells were treated for 24 or 48 h with 0.1% DMSO vehicle control or 0, 0.1, 1, 5 or 10 µM of pyrazinib (P3), after which time they were transferred to a FACS tube and washed in 1 mL 1X binding buffer. Cells were stained with 3 μl of Annexin V antibody and incubated in the dark for 15–20 min at 4 °C. Cells were washed and resuspended in 250 μL of 1 in 4,000 dilution of PI in 1X binding buffer and samples were immediately acquired on FACSCanto II flow cytometer (BD Biosciences).

### Assessing the activation status of Jurkat cells

Unactivated and activated Jurkat cells were seeded 5 × 10^4^ cells/mL in 12 well plates in complete RPMI. Jurkats were activated with plate bound anti-CD3 (Biolegend, CA, USA) and anti-CD28 (Ancell, MN, USA) monoclonal antibodies for 24 h. Cells were treated for 24 or 48 h with 0.1% DMSO vehicle control or 10 µM of pyrazinib (P3). Cells were washed and blocked and then stained with anti-CD62 PerCp-Cy5 (Abcam, UK), anti-CD69 PE (BD Biosciences, CA, USA), anti-CD45RO FITC (Immunotools, Germany), anti-CD45RA V500 (BD Biosciences) and anti-CD27 APC eFluor780 (eBioscience. CA, USA) for 20 min at 4 °C in the dark. Cells were washed and resuspended in 300 μL FACS buffer and acquired on a FACSCanto II flow cytometer (BD Biosciences).

### Statistical analysis

Statistical analysis was performed using GraphPad Prism software version 5 (GraphPad Software, CA, USA). Scientific data were expressed as mean ± SEM. Specific statistical tests used are indicated in figure legends. For all statistical analysis, differences were considered to be statistically significant at *p* < 0.05.

## Supplementary information

Supplementary information
