# Supplementary material for: Real-time metabolic profiling of oesophageal tumours reveals an altered metabolic phenotype to different oxygen tensions and to treatment with Pyrazinib
Source: Sci Rep. 2020 Jul 21;10:12105. doi: 10.1038/s41598-020-68777-7 (PMC7374542; doi:10.1038/s41598-020-68777-7)
Supplement: Supplementary file 1 — Supplementary information [file 41598_2020_68777_MOESM1_ESM.pdf]

Real-time metabolic profiling of oesophageal tumours reveals an altered metabolic phenotype to different oxygen tensions and to treatment with Pyrazinib

Amy M. Buckley[1], Margaret R. Dunne[1], Maria E. Morrissey[1], Susan A. Kennedy[1], Aoife Nolan[1], Maria Davern[1], Emma K. Foley[1], Niamh Clarke[1], Joanne Lysaght[1], Narayanasamy *Ravi*[1], Dermot O'Toole[2], Finbar MacCarthy[2], John V. Reynolds[1], Breandán N. Kennedy[3], Jacintha O'Sullivan[1*].

[1]Department of Surgery, Trinity Translational Medicine Institute, St. James's Hospital, Trinity College Dublin, Ireland.

[2]Department of Clinical Medicine, Trinity Translational Medicine Institute, St. James's Hospital, Trinity College Dublin, Ireland.

[3]UCD Conway Institute & UCD School of Biomolecular and Biomedical Science, University College Dublin.

Contact Information: Correspondence to Prof. Jacintha O'Sullivan, osullij4@tcd.ie

Supplemental Material

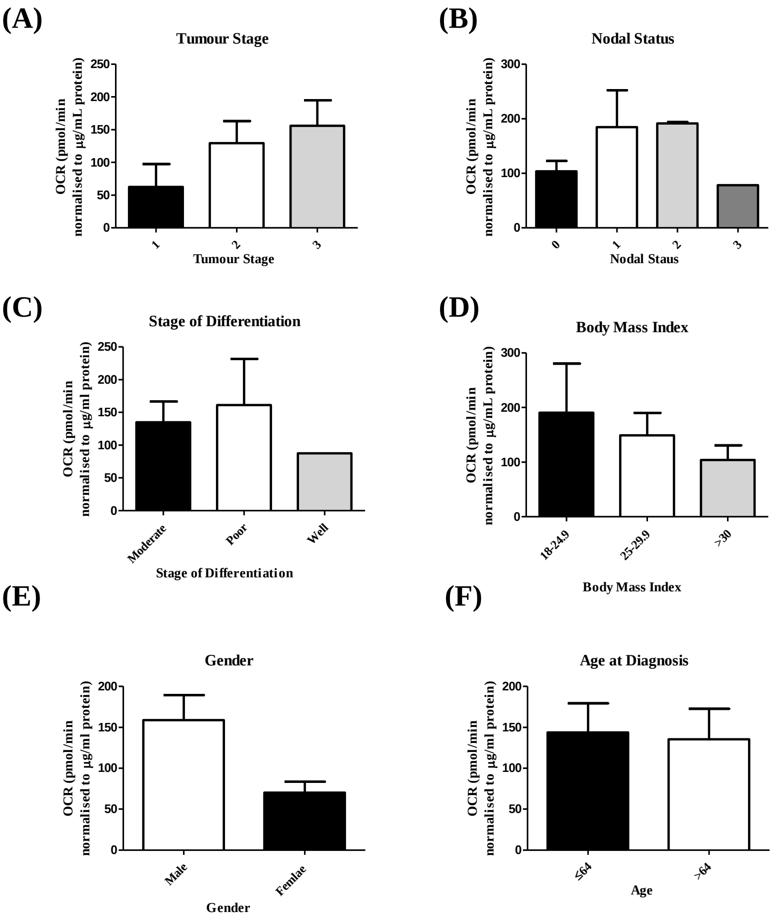

Supplemental Figure 1. Real-time oxygen consumption rate is not significantly associated with clinical patient characteristics in OAC pre-treatment biopsies.

Baseline OCR readings of OAC treatment-naïve biopsies divided according to **(A)** Tumour Stage (n=16), **(B)** Nodal Status, (n=17), **(C)** Stage of Differentiation, (n=16), **(D)** Body Mass Index, (n=16), **(E)** Gender (n=17) and **(F)** Age at Diagnosis, (n=17). Mann Whitney U test. Data expressed as +SEM.

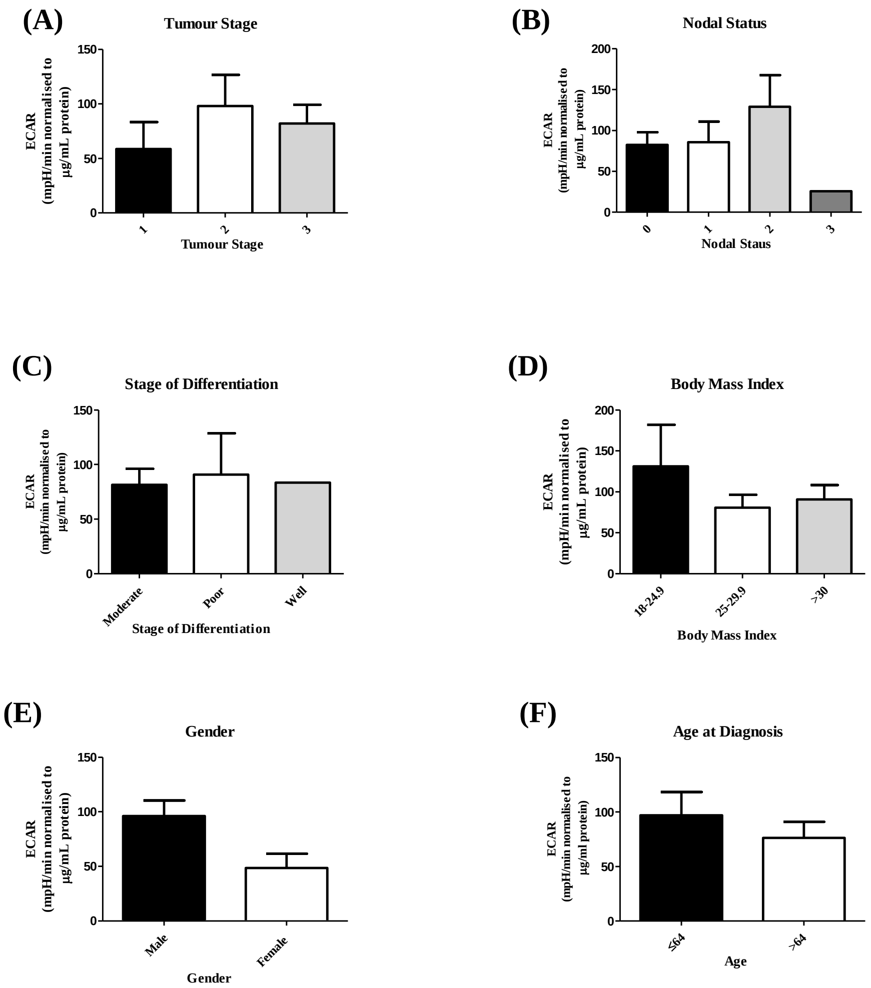

Supplemental Figure 2. Real-time extracellular acidification rate is not significantly linked with clinical patient characteristics in OAC pre-treatment biopsies.

Baseline ECAR readings of OAC treatment-naïve biopsies divided according to **(A)** Tumour Stage (n=16), **(B)** Nodal Status, (n=17), **(C)** Stage of Differentiation, (n=16), **(D)** Body Mass Index, (n=16), **(E)** Gender (n=17) and **(F)** Age at Diagnosis, (n=17). Mann Whitney U test. Data expressed as +SEM.

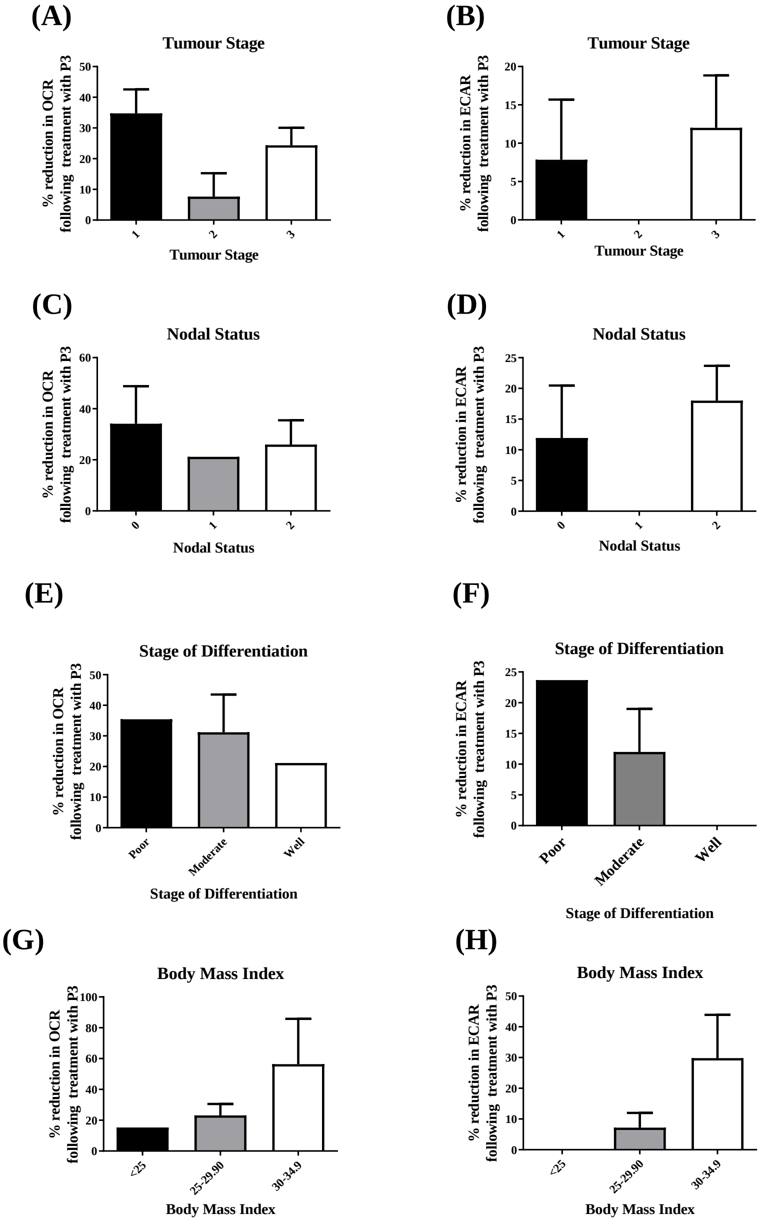

Supplemental figure 3.The anti-metabolic activity of Pyrazinib (P3) is not dependant on clinical patient characteristics.

OCR and ECAR were measured in real-time using Seahorse Biosciences XFe24 analyser. Percentage reduction of OCR following 24 h treatment with P3 was divided according to **(A)** Tumour Stage, (n=9), **(C)** Nodal Status (n=9), **(E)** Stage of differentiation (n=9), and **(G)** Body Mass Index (n=9). Percentage reduction of ECAR following 24 h treatment with P3 was divided according to **(B)** Tumour Stage, (n=9), **(D)** Nodal Status, (n=9), **(F)** Stage of differentiation, (n=9) and **(H)** Body Mass Index, (n=9). Mann-Whitney U test. Data are expressed as +SEM.

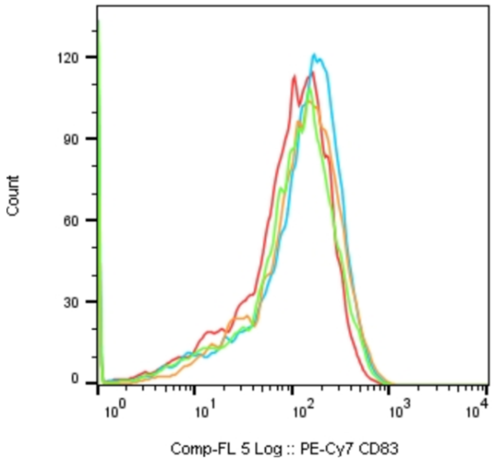

Sample	Subset	Count
0.1% DMSO + M199 Media	CD11c$^+$	6100
Pyrazinib (P3)+ M199 Media	CD11c$^+$	6149
TCM + 0.1% DMSO	CD11c$^+$	6608
TCM + Pyrazinib (P3)	CD11c$^+$	6813

Supplemental Figure 4. Representative histogram of CD83 expression in CD11c+ cells
Representative histogram illustrating changes in MFI in CD83 expressing CD11c+ cells following stimulation with LPS.

Supplemental Table 1. Clinical patient characteristics of patient cohort used in normoxia metabolism study.

Table detailing the clinical patient characteristics of the 10 patients whose biopsies were used to investigate the effective of Pyrazinib (P3) on real-time metabolic rate under normoxic conditions.

	**n=10**
**Male**	8
**Female**	2
**Median age at diagnosis**	70.5 (57-80)
**Median BMI at diagnosis**	27.7 (24.3-30.8)
**Tumour Stage**	
1	2
2	3
3	4
Not Specified	1
**Nodal Status**	
0	7
1	1
2	2
**Stage of Differentiation**	
Poor	3
Moderate	5
Well	1
Not Specified	1
**Tumour Regression Grade**	
3	4
4	1
Not specified	5

Supplemental Table 2. Clinical patient characteristics of patient cohort used in hypoxia metabolism

This table details the clinical patient characteristics of the 7 patients whose biopsies were used to investigate real-time metabolic rate under normoxic conditions. (Only 6 of these patients' biopsies were used to evaluate the effect of Pyrazinib (P3) on real-time metabolic rate under hypoxic conditions due to limited patient material for one patient).

	**n=7**
**Male**	7
**Female**	0
**Median Age at diagnosis**	68 (54-83)
**Median BMI at diagnosis**	27.2(21.2-30)
**Tumour Stage**	
3	7
**Nodal Status**	
0	2
1	4
3	1
**Stage of Differentiation**	
Poor	2
Moderate	5
**Tumour Regression Grade**	
1	1
2	1
3	0
4	1
5	1
Not specified	3

Supplemental Table 3. Clinical patient characteristics of patient cohort used in multiplex ELISA and dendritic cell study.

This table details the clinical patient characteristics of the 22 patients whose biopsies were used to investigate the inflammatory secretions from control and Pyrazinib (P3) treated TCM.

	**n=22**
**Male**	18
**Female**	4
**Median age at diagnosis**	67 (43-84)
**Median BMI at diagnosis**	27.9 (23.5-38.5)
**Tumour Stage**	
1	3
2	5
3	12
4	1
Not Specified	1
**Nodal Status**	
0	7
1	11
2	4
**Stage of Differentiation**	
Poor	9
Moderate	10
Well	2
Not Specified	1
**Tumour Regression Grade**	
1	1
2	0
3	6
4	2
5	2
Not specified	11
